# Exploration of barriers and enablers for the use of the nutrition care process among a diverse sample of registered dietitian nutritionists: a mixed methods analysis

**DOI:** 10.3389/fnut.2026.1727518

**Published:** 2026-01-29

**Authors:** Irene A. Asare, Constantina Papoutsakis, Lauri Wright, Casey Colin

**Affiliations:** 1 Department of Nutrition and Dietetics, University of North Florida, Jacksonville, FL, United States; 2 Data Science Center, Research, International, and Scientific Affairs (RISA), Academy of Nutrition and Dietetics, Chicago, IL, United States; 3 Nutrition Programs, College of Public Health, University of South Florida, Tampa, FL, United States

**Keywords:** barriers, dietitian, education, enablers, nutrition care process, nutrition care process terminology

## Abstract

**Background:**

Registered dietitian nutritionists (RDNs) use the Nutrition Care Process and its Terminology (NCP/T) to generate outcomes and demonstrate the impact of medical nutrition therapy. Despite integration into education in 2009, many RDNs continue to face challenges in its application.

**Objective:**

This study aimed to identify barriers and enablers to NCP/T use to better understand how adoption can be improved, and to assess whether qualitative feedback from practicing RDNs aligns with quantitative findings from the 2017 International Nutrition Care Process and Terminology Implementation Survey (INIS).

**Design:**

An explanatory sequential mixed-methods approach was used. Quantitative data was from United States based RDNs who participated in the 2017 INIS. The focus group discussion questions were informed by the INIS study and grounded in the Theory of Planned Behavior (13). Zoom technology (14) was used and all the discussions were audio recorded. Only participants and the interviewer were present on the call. Qualitative data from the focus group discussions included RDNs in clinical, community, and academic settings. Semantic thematic analysis was conducted to identify themes related to barriers and enablers to using NCP/T.

**Participants/setting:**

INIS study recruitment utilized email lists, e-newsletters, and social media groups; responses from 4,426 active RDNs were analyzed. Focus group inclusion criterion was active RDNs based in the US; 38 RDNs participated in the focus groups.

**Statistical analyses performed:**

Cross-tabulation identified correlations between barriers/enablers and characteristics such as years of practice and practice setting (*p* < 0.05).

**Results:**

INIS data showed an association between practice area, years of experience, and NCP/T use (*p* < 0.001). Enablers included peer support (59% of clinical RDNs, 60.3% of RDNs with 0–5 practice years) and job requirements (52.9% of clinical RDNs, 55.2% of those with 0–5 years). Barriers included limited time (28.9% of clinical RDNs, 29.4% with >16 years) and insufficient education (25% of clinical RDNs, 29.8% with >16 years). Focus groups identified additional enablers, such as integrating NCP/T into Electronic Health Records, and barriers, including eNCPT subscription access.

**Conclusion:**

The INIS study and focus groups revealed consistent barriers and enablers, underscoring the need for authoritative state-of-the-art training to address these factors and enhance NCP/T utilization.

## 1 Introduction

The Nutrition Care Process (NCP) was adopted in 2003 by the Academy of Nutrition and Dietetics (Academy) to provide RDNs with a standardized process for delivering quality and individualized nutrition care with predictable outcomes (1). To support the four steps of the NCP, the Nutrition Care Process Terminology (NCPT) was introduced as a set of standardized language in 2009 (1). The use of the Nutrition Care Process and Terminology (NCP/T) allows researchers to identify data that may demonstrate the relationship between Medical Nutrition Therapy (MNT) and nutrition outcomes (1, 2). Additionally, standardized terminology offers a framework for consistent dietetics practice while also supporting justification for RDNs’ contributions to improved health outcomes and their necessity within the interdisciplinary medical team. NCP is integral to the educational standards for RDNs in the United States (U.S). It outlines a sequence of essential steps, including assessment, diagnosis, intervention, and monitoring/evaluation, designed to ensure that patients receive the highest quality of nutrition care tailored to their unique needs (1). The Academy mandates NCP as a professional standard for RDNs (1, 2). The expectation within the profession is that all RDNs in the U.S. should be utilizing the NCP as a fundamental component of their practice. However, the degree of implementation may vary among practitioners and facilities (3).

Although NCP was developed in the U.S and first adopted by the Academy, its use has expanded internationally over the past two decades. Several countries, including Canada (4), the United Kingdom, Australia, and parts of Europe, have adopted the NCP/T to promote standardized nutrition care and documentation (5, 6). In some countries, such as Canada and Australia, use of the NCP is strongly encouraged by professional dietetic associations and embedded within education and practice standards, though not universally mandated (4). In others, including selected European and Asian countries, implementation varies by setting and is often driven by institutional policy, or research initiatives rather than national requirements (5–9). This growing global uptake underscores the NCP’s relevance as an international framework for delivering and documenting nutrition care, while also highlighting the need for context-specific training and support to promote consistent and effective use across diverse health systems.

Research studies show that RDNs in most countries continue to encounter barriers in implementing and utilizing the NCP/T, which limits data collection vital to outcomes research (4–9). In 2017, the International Nutrition Care Process and Terminology Survey (INIS) was created to investigate the barriers and enablers encountered by nutrition and dietetic experts when implementing NCP (5). The survey was administered to a total of 6,719 RDNs who resided in 10 different countries, including the U.S. Practitioners reported “recommendation by the national dietetic association,” “peer support,” and the “use of Electronic Health Records (EHR),” as the greatest enablers. The barriers reported included “lack of time,” “education,” “training,” and “knowledge.” Studies in Saudi Arabia (7) and South Korea (9) have also investigated barriers and enablers to NCP/T adoption. In Saudi Arabia, 95% of 56 RDNs were aware of NCP, but only 27% received formal training, and 26% used NCPT. Key barriers included limited staff and insufficient knowledge or experience (7). In South Korea, 90% of clinical nutrition managers from 35 hospitals were familiar with NCP, 75% had training, but only 25% implemented it, citing lack of knowledge and time as primary barriers (9). These international studies provide valuable insights and context that help to understand the global barriers and enablers of NCP implementation, demonstrating that despite the 2002 adoption of NCP by the Academy, challenges persist for RDNs globally (4–9).

It is important to note that, to the best of our knowledge, no existing studies exclusively examine barriers and enablers among RDNs solely based in the U.S. The INIS study included U.S. Participants but did not exclusively focus on them (5). This study aims to fill this gap by focusing specifically on U.S.-based RDNs who participated in the 2017 INIS study and also utilized focus group discussions conducted for this research to explore barriers and enablers to using NCP/T.

## 2 Methods

### 2.1 INIS study

This explanatory sequential mixed-method design (10) included a quantitative component, which involved secondary data analysis of 4,426 U.S.-based RDNs who participated in the 2017 INIS study. The INIS study aimed to evaluate how the NCP/T was being implemented globally. Using a validated web-based survey, the study gathered information on enablers and barriers to using NCP/T. The study was approved by the Uppsala, Sweden, ethics review board (Dnr 2016/258). Participants provided written informed consent. Email lists, e-newsletters, and RDN social media groups were used for recruitment. The survey excluded non-RDNs and took 20 min to complete. The survey comprised of four modules: demographic characteristics (Module 1), NCP/T implementation (Module 2), NCP/T attitudes (Module 3), and NCP/T knowledge (Module 4). The data utilized for the current study focused on demographic characteristics, enablers, and barriers to NCP/T utilization that were reported by the U.S.-based participants (5).

Quantitative analysis was conducted using SPSS (version 29) (11). Descriptive statistics were used to summarize participant characteristics, while cross-tabulations were used to examine associations between barriers/enablers and demographic characteristics such as years of practice and practice settings. Statistical significance was set at *p* < 0.05. *Post hoc* analyses were conducted to examine which barriers and enablers differed significantly by years of practice (0–5 years, 6–15 years, and ≥16 years).

### 2.2 Focus groups

The focus group study and secondary data analysis of INIS was approved by the University of North Florida Institutional Review Board # 1956316-3. Focus group participants completed written consent. The population for the focus group discussions included U.S.-based RDNs in clinical, community, or education (academia). Given the diverse settings in which RDNs practice, we sought to capture the broadest range of practice areas. Inclusion criteria were: (a) active RDNs with a current credential from the Commission on Dietetic Registration (CDR), and (b) practicing in outpatient, inpatient, long-term care, private practice, community-based settings, or teaching in academia.

A list of U.S.-based RDNs (*N* = 95,708) was obtained from CDR, and 5.73% (*N* = 5,484) were selected using stratified sampling. Participants were then sent an email that included the aims and reasons for conducting the research, the study’s flyer, and a link to a survey. Respondents were categorized by years of practice and practice area: clinical and community RDNs were divided into 0–10 years and 11+ years, while RDNs in academia were grouped into 0–15 years and 16+ years to ensure sufficient responses. Thus, focus group discussions were conducted for six distinct groups of RDNs: (a) clinical RDNs with less than 10 years of practice, (b) clinical RDNs with more than 10 years of practice, (c) community RDNs with less than 10 years of practice, (d) community RDNs with more than 10 years of practice, (e) education RDNs with less than 15 years of practice, and (f) education RDNs with more than 15 years of practice. Each group of the six groups consisted of 7–12 participants.

A trained researcher, C.C., with qualitative research experience from her doctoral program, conducted focus group discussions with 38 RDNs who responded to the email invitation. Although the focus group guide was not pilot tested, it was theoretically grounded and subjected to both face and content validation by an expert in NCP and an expert in qualitative research, thereby enhancing its methodological rigor. Semantic thematic analysis (12) was conducted to identify themes related to barriers and enablers to using NCP/T. The sample size of 38 dietitians was sufficient to achieve data saturation, ensuring that no new themes emerged with additional discussions. Participants represented eight U.S. Regions, enhancing geographic diversity and providing varied perspectives on NCP/T adoption. While the sample prioritized depth over breadth, the diversity of practice settings and experience levels allowed for a comprehensive exploration of barriers and enablers to NCP/T use among RDNs. The discussion questions were informed by the INIS study and grounded in the Theory of Planned Behavior (13). Zoom technology (14) was used and all the interviews were audio recorded. Only participants and the interviewer were present on the call. Transcripts were used for analyses to explore participants’ insights.

Qualitative analysis of focus group discussions was conducted using NVivo (version 12) (15) for data transcription and organization. Two researchers (C.C. and I.A.) independently coded transcripts, resolving discrepancies through discussion. New codes were added iteratively, and all codes were reviewed and refined to capture emerging themes. A thematic list was developed and finalized to ensure it reflected the main concepts and recurring patterns from the interviews.

## 3 Results

### 3.1 Quantitative analyses (INIS data)

#### 3.1.1 Sample characteristics

The quantitative data derived from the 2017 INIS study comprised of 4,426 active RDNs in the U.S. who used NCP/T. Of these, 912 (20.6%) RDNs reported practicing in community settings, while 3,514 (79.4%) practiced in clinical settings. Additionally, 1,653 (37.3%) had 0–5 years of experience whereas 1,499 (33.9%) and 1,260 (28.5%) had 6–15 and over 16 years of practice experience, respectively. A total of 2,252 (50.9%) held a graduate degree and 2,169 (49%) held a bachelor’s degree. The majority of the participants, 4,272 (96.5%) had heard of NCP but 146 (3.3%) had not.

#### 3.1.2 Barriers to using NCP/T

Table 1 presents the association between area of practice and reported barriers to the use of the NCP/T among RDNs. Significant associations were identified between area of practice and the following barriers: lack of time, lack of motivation, and electronic health record (EHR) unavailability. Specifically, a greater proportion of RDNs in clinical settings (28.9%) reported lack of time as a barrier compared with those in community settings (22.0%) (*p* < 0.001). Similarly, lack of motivation was reported more frequently by RDNs in clinical settings (22.0%) than by those in community settings (16.7%) (*p* < 0.001). In contrast, EHR unavailability was reported more often by RDNs in community settings (13.7%) than by those in clinical settings (11.4%) (*p* < 0.05). No other significant associations were observed between area of practice and the remaining barriers, suggesting that these barriers were reported similarly across practice settings.

**Table 1:** Barriers to using NCP/T stratified by RDNs’ area of practice.

Barriers	Community *n* (%)	Clinical *n* (%)	*p*-value
Lack of time	201 (22.0%)	1,014 (28.9%)	<0.001
Lack of peer support	116 (12.7%)	502 (14.3%)	NS^a^
Not having access to online tools or books	124 (13.6%)	513 (14.6%)	NS^a^
Lack of motivation/do not see a reason to change my work approach	152 (16.7%)	772 (22.0%)	<0.001
Lack of management support	179 (19.6%)	738 (21%)	NS^a^
Lack of financial resource	98 (10.7%)	393 (11.2%)	NS^a^
Electronic healthcare records are unavailable	125 (13.7%)	399 (11.4%)	0.05
Lack of training and education	214 (23.5%)	878 (25.0%)	NS^a^
Lack of knowledge	179 (19.6%)	770 (21.9%)	NS^a^

a = non-significant.

When stratified by years of practice, the most frequently reported barrier among RDNs with less than 5 years of experience (24.6%) and those with 6–15 years of experience (29.1%) was lack of time to use NCP/T. Among RDNs with more than 16 years of practice, lack of training was the most frequently reported barrier. Cross-tabulation analyses showed that these barriers were significantly associated with years of practice (*p* < 0.05). Post-hoc analyses revealed that for lack of time, lack of motivation to change work approach, lack of financial resources, lack of training and education, and lack of knowledge, RDNs practicing for 0–5 years differed significantly from those practicing for 6–15 years and ≥16 years (Table 2). No significant differences were observed between the 6–15 years and ≥16 years groups for these barriers, indicating that the observed effects were driven primarily by lower barrier reporting among early-career RDNs.

**Table 2:** Barriers to using NCP/T stratified by RDNs’ years of practice.

Barriers	0–5 years *n* (%)	6–15 years *n* (%)	>16 *n* (%)	*p*-value
Lack of time^b,c^	406 (24.6%)	436 (29.1%)	371 (29.4%)	0.003
Lack of peer support	225 (13.6%)	231 (15.4%)	162 (12.9%)	NS^a^
Not having access to online tools or books^d^	243 (14.8%)	234 (15.6%)	159 (12.6%)	NS^a^
Lack of motivation/do not see a reason to change my work approach^b,c^	214 (12.9%)	405 (27%)	303 (24%)	0.001
Lack of management support	354 (21.4%)	306 (20.4%)	254 (20.2%)	NS^a^
Lack of financial resource^b,c^	143 (8.7%)	174 (11.6%)	171 (13.6%)	<0.001
Electronic healthcare records are unavailable	189 (11.4%)	169 (11.3%)	166 (13.2%)	NS^a^
Lack of training and education^b,c^	278 (16.8%)	438 (29.2%)	375 (29.8%)	<0.001
Lack of knowledge^b,c^	226 (13.7%)	400 (26.7%)	322 (25.6%)	<0.001

a = non-significant.

b = post hoc test denoting significant differences between 0–5 years and 6–15 years.

c = post hoc test denoting significant differences between 0–5 years and 16 years and above.

d = post hoc test denoting significant differences between 6–15 years and 16 years and above.

#### 3.1.3 Enablers to use NCP/T

All nine enablers were significantly associated with area of practice, with a higher proportion of clinical RDNs reporting each enabler compared to community RDNs (Table 3) (*p* < 0.05). Similarly, RDNs with 0–5 years of experience reported each enabler at significantly higher rates than those with more years of practice (Table 4). The recommendation of NCP/T by the Academy was the most frequent enabler for RDNs irrespective of their area or years of practice. This was followed by peer support and the incorporation of EHR. Post-hoc analyses indicated significant differences in most enablers across years of practice. For peer support, allocated time to practice, designated leader, and professional association recommendation, RDNs practicing ≥16 years reported significantly lower endorsement than both those practicing 0–5 years and 6–15 years (*p* < 0.001). For management support, electronic health records, workplace requirement, supervision of students, and professional association recommendation, all three groups differed significantly, with endorsement decreasing progressively with increasing years of practice. Regular education and training sessions differed significantly between RDNs practicing 0–5 years and both 6–15 years and ≥16 years, but not between the two more experienced groups.

**Table 3:** Enablers to using NCP/T stratified by RDNs’ area of practice.

Enablers	Community *n* (%)	Clinical *n* (%)	*p*-value
Peer support	369 (40.5%)	2,075 (59.0%)	<0.001
Allocated time to practice	276 (30.3%)	1,462 (41.6%)	<0.001
Management support	277 (30.4%)	1,617 (46.0%)	<0.001
Electronic healthcare records	326 (35.7%)	2,119 (60.3%)	<0.001
Required by my workplace	231 (25.3%)	1,859 (52.9%)	<0.001
Regular education and training sessions	320 (35.1%)	1,414 (40.2%)	0.005
Designated leader at the workplace	247 (27.1%)	1,313 (37.4%)	<0.001
Required when supervising dietetic students	273 (29.9%)	1,679 (47.8%)	<0.001
Recommended by the professional dietetic association	450 (49.3%)	2,363 (67.2%)	<0.001

**Table 4:** Enablers to using NCP/T stratified by RDNs’ years of practice.

Enablers	0–5 years *n* (%)	6–15 years *n* (%)	>16 years *n* (%)	*p*-value
Peer support^b,c^	996 (60.3%)	865 (57.7%)	576 (45.7%)	<0.001
Allocated time to practice^b,c^	822 (49.7%)	555 (37.0%)	356 (28.3%)	<0.001
Management support^a,b,c^	811 (49.1%)	665 (44.4%)	413 (32.8%)	<0.001
Electronic healthcare records^a,b,c^	1,122 (67.9%)	792 (52.8%)	526 (41.7%)	<0.001
Required by my workplace^a,b,c^	912 (55.2%)	710 (47.4%)	462 (36.7%)	<0.001
Regular education and training sessions^a,b^	757 (45.8%)	559 (37.3%)	412 (32.7%)	<0.001
Designated leader at the workplace^b,c^	667 (40.4%)	535 (35.7%)	354 (28.1%)	<0.001
Required when supervising dietetic students^a,b,c^	882 (53.4%)	673 (44.9%)	391 (31%)	<0.001
Recommended by the professional dietetic association^a,b,c^	1,228 (74.3%)	928 (61.9%)	651 (51.7%)	<0.001)

a = post hoc test denoting significant differences between 0–5 years and 6–15 years.

b = post hoc test denoting significant differences between 0–5 years and 16 years and above.

c = post hoc test denoting significant differences between 6–15 years and 16 years and above.

### 3.2 Qualitative analyses (focus group data)

#### 3.2.1 Sample characteristics

A total of 38 RDNs responded and completed the focus group discussions. Of the 38 RDNs, 55% (21) worked as clinical RDNs, 23% (9) as community RDNs, and 21% (8) were educator RDNs. Ten participants in total had less than 10 years of work experience, with 3 participants each from the clinical and community practice groups. In the education group, 4 participants had more than 15 years of experience, while another 4 had less than 15 years. The interviews ranged in duration from 42 to 83 min, with an average length of 64 min.

#### 3.2.2 Barriers to using NCP/T

Five major themes regarding barriers to using NCP/T emerged from the interviews: (1) limited knowledge; (2) time-consuming; (3) adds no value to notes; (4) NCP is not built into EHR; and (5) no access to eNCPT (the electronic edition of NCPT). Theme descriptions and supportive quotes are detailed below and in Table 5.

**Table 5:** Themes, descriptions, and representative quotes on barriers to using NCP/T among 38 RDNs in 6 focus groups.

Themes	Description	Supportive quotes
Limited knowledge	Poor or limited knowledge of the use, benefits and purpose of NCP/T	“…Dietitians my age who were not taught it have a hodgepodge understanding of it, and then you have your PES that whoever developed them and their understanding of it is a whole other situation. So, it’s kind of all over the place. And then understanding how it’s utilized…”
Time-consuming	NCP/T use increases charting time	“…I do think that it is very overwhelming when you look at the notes, and that’s sort of the feedback that I get. We get the PES statement. But all the other stuff like is just sort of we do not have the time to sit down and put all the standardized terminology into everything, and put the numbers with everything…”
Adds no value to notes	Lack of motivation to change	“…I think it makes us look stupid.”
No access to eNCPT	Inability to access eNCPT due to budgeting constraints	“…We used to have little spiral bound books. Now there is paid access to everything, and it’s very difficult…”
NCP is not built into EHR	Existing EHR does not allow for the addition of NCP/T	“…even if I think we wanted to use it in my company, I do not even think we would be able to, because of the way the EHR is set up…”

##### 3.2.2.1 Theme 1: limited knowledge

Several RDNs, particularly those with over 10 years of experience, reported a limited understanding of NCP/T. Some found it unnatural to use, citing challenges like, *“*I do not use related to and as evidenced by when I speak.” They also noted communication barriers, as their documentation was often misunderstood or ignored by colleagues, hindering collaboration and patient care.

##### 3.2.2.2 Theme 2: time consuming

A common theme among participants was that the NCP is time-consuming, and using NCPT increased workload, particularly among clinical RDNs with less than 10 years of experience. One participant stated, “Using NCP makes charting more difficult,” reflecting a broader sentiment among this group. In contrast, RDNs with over 10 years of experience preferred the faster, more familiar Subjective, Objective, Assessment, and Plan (SOAP) notes format, which they felt was better understood by the interdisciplinary team (IDT).

##### 3.2.2.3 Theme 3: no value

Most community RDNs felt the NCP format added little value to their notes. Many found NCP limiting, citing difficulties in individualizing care and summarizing nutritional problems with a single PES statement, often using it merely “to go through the motions.” The lack of recognition and support for NCP from IDT further discouraged its use and integration.

##### 3.2.2.4 Theme 4: not built into the EHR system

Another barrier was the absence of NCPT in EHR systems. Participants facing this issue included those in private practice and small facilities still using paper charts or outdated EHR systems. One RDN voiced “everybody wants EPIC or something equivalent to EPIC, and EPIC itself is a very expensive program. So that’s why some folks do not have the drop-down options.”

##### 3.2.2.5 Theme 5: no access to eNCPT

The absence of access to eNCPT emerged as a barrier, hindering RDNs’ ability to incorporate standardized terminology into their notes. A comment such as “many of us do not have organizations that pay for eNCPT subscription, the charge is too much.” was made to depict the financial burden in accessing eNCPT.

#### 3.2.3 Enablers to use NCP/T

The five major themes regarding enablers to using NCP/T that emerged from the interviews were: (1) it helps with communication; (2) it’s required by CMS; (3) teach NCP to students; (4) it’s built into the EHR systems; and (5) it’s required by the job or by the Academy. Theme descriptions and supportive quotes are detailed in Table 6.

**Table 6:** Themes, descriptions, and representative quotes on enablers to using NCP/T among 38 RDNs in 6 focus groups.

Themes	Description	Supportive quotes
It helps with communication	It fosters communication between RDNs and IDT members	“…I think it’s more reliable when you take a patient that transfers from a hospital, at least you know what to look for because it’s been standardized…”
It’s required by CMS	It is required for coding and billing	“…I think it’s been helpful, especially in terms of a nutrition diagnosis and helping with billing and coding…”
Teach NCP to students	RDNs use it to gain a sufficient understanding that would enable them to teach it to dietetic interns.	“…I’m a preceptor for a lot of interns during the dietetic internship, so I have to make sure that I’m following it in order to give them good examples of how to properly counsel, and so I find that it’s definitely helpful to learn the sessions…”
It’s built into the EHR systems	NCP is already built into the EHR systems, thus making it readily available	“…Our agency would probably still not be using this if it wasn’t automatically programmed into this electronic charting system…”
It’s required by the job or the Academy	It is a job requirement	“…Yeah, it’s part of our job requirement. So that’s why I use it…”

##### 3.2.3.1 Theme 1: helps with communication

Participants highlighted NCPT’s role in improving communication among RDNs by providing a standardized language and framework for conveying nutritional information. This feature was seen as a key enabler of collaboration, fostering a shared understanding. One participant noted, “…I think it helps coordinate care when we pass notes to other providers.”

##### 3.2.3.2 Theme 2: it is required by CMS

The necessity of the NCP/T for coding and billing purposes emerged as an enabler. RDNs recognized the importance of adhering to coding and billing requirements, viewing the NCP as a valuable tool in ensuring accurate and compliant documentation for financial and reimbursement purposes. One participant stated “…sometimes people deal with CMS, you know, Medicaid. They have to put in those criteria, you know, what’s covered so it is helpful.”

##### 3.2.3.3 Theme 3: teach NCP to students

The requirement to use the NCP in the context of mentoring and training future RDNs served as a compelling enabler, emphasizing its role not only as a professional obligation but also as an educational tool. One participant remarked “… I’ve had interns coming from all over, and part of them will use that. So, I needed to learn it more, so that I could make sure that I was giving them correct information, too.”

##### 3.2.3.4 Theme 4: it is built into the EHR system

The fourth enabler was integrating NCP/NCPT into EHR systems, which RDNs appreciated for streamlining workflows. Some noted it was mandatory for documentation. One participant shared, “…so we use an EHR, and it’s all built in. There are boxes. more writing within the boxes. And those boxes. have headings just like a normal ADIME.”

##### 3.2.3.5 Theme 5: it is required by the job or the academy

A recurrent theme, especially among clinical RDNs with less than 10 years of experience, was that using NCP is a mandatory job requirement. When asked, “What has forced you to use NCP?” most cited their job and the Academy’s endorsement. One RDN remarked, “…if you want to keep your certification, if you want to be accredited by CDR, you better be using it.”

### 3.3 How can the academy help?

During the focus group interviews, participants were asked what the Academy can do to help increase the use of NCP/T. The three themes that emerged were free educational materials, leadership support and categorization of NCPT by specialty area of practice. However, the predominant theme was the request for the Academy to provide free educational tools.

## 4 Discussion

This study is the first to combine quantitative and qualitative data to examine barriers and enablers for RDNs using NCP/T, while also exploring their correlation with years and areas of practice. Combining findings from the INIS study and focus group discussions, identified barriers included time, training, motivation, financial resources, and limited access to tools. Enablers included peer support, job or Academy adoption, communication assistance, precepting, and integration of NCP/T into EHR. While consistent with prior research (4–6), no previous studies have linked barriers and enablers to RDNs’ specialization and practice experience.

### 4.1 Barriers based on area of practice

#### 4.1.1 Barrier 1: lack of time

The statistically significant correlation (*p* < 0.001) between the area of practice and time constraints shown in the INIS data suggests that RDNs in some practice settings may be more burdened by workload expectations, leaving them with insufficient time to adequately explore and use NCP/T. In both the INIS data and the focus group discussions, this barrier was very common among those who practiced in the clinical setting. This finding is consistent with other studies that show time constraints are typical obstacles in clinical settings with high levels of intensity (5, 17). Thus, to support the incorporation of NCP/T in these contexts, time management techniques or workflow modifications may be required. Adopting the use of EHR systems (18), concise note templates and standardized acronyms have been listed as practices for RDNs to save time (19).

#### 4.1.2 Barrier 2: lack of motivation

The significant association between practice area and lack of motivation to use NCP/T (*p* = 0.001) highlights varying perceptions of its value across settings. In the INIS study, 16.7% of community RDNs and 22% of clinical RDNs reported this barrier, with community-based RDNs citing it most often in the focus group discussions. Some RDNs may not see the benefits of standardized procedures, thereby favoring flexible care methods. Similarly to these findings, O’Sullivan et al. (8) identified “not seeing a reason to change” as a barrier among Asia-Pacific RDNs but did not link it to practice areas. These findings stress the need to promote NCP/T benefits through education, leadership support, and tailored performance objectives to boost motivation in low-engagement settings.

#### 4.1.3 Barrier 3: unavailability of EHR systems

Given the importance of EHR systems to the successful implementation of NCP/T, the correlation between practice areas and the unavailability of EHR as a barrier is alarming (*p* = 0.05). Similar findings were reported in a Canadian study (4) in which the authors noted that the adoption of EHR systems may lag behind larger institutions in smaller or resource-constrained areas, such as private practices or rural health clinics, where this barrier may be more noticeable. The benefits of NCP/T in enhancing patient outcomes may be limited if EHR systems are not available, making it difficult to standardize the documentation and track nutrition care. This barrier was identified by clinical RDNs in the focus group discussions as well as community RDNs in the INIS study (13.7%), indicating a consistent link between the barrier and the area of practice. Therefore, investing in EHR systems is essential to help RDNs across all practice settings overcome this challenge.

### 4.2 Barriers based on years of practice

The INIS data also showed a significant correlation between the years of practice and the following barriers: lack of time, motivation, financial support, education, and knowledge.

#### 4.2.1 Barrier 1: lack of time

RDNs at different phases of their careers may face varied amounts of time constraints, as seen by the association between years of practice and the lack of time. Proficient and expert RDNs with more expertise and a heavier caseload may find it more difficult to set aside time for the thorough documentation and patient assessment that NCP/T requires. On the other hand, entry-level RDNs may find it difficult to manage their time because they are still honing their clinical efficiency. Time restriction has been noted in previous studies as a common barrier in healthcare (20). It also implies that approaches to this problem might need to be customized based on the career stage of the RDN.

#### 4.2.2 Barrier 2: lack of motivation

Practice inertia is a phenomenon that details the challenges of encouraging behavior change among seasoned professionals (16, 21). RDNs with extensive experience who are firmly rooted in traditional methods of documentation may find it harder to embrace new processes and terminologies. This barrier was also evident among the focus group participants who had been in practice for over 10 years. RDNs in that group suggested a more personalized and adaptable approach to the NCP/T which indicates that the current structure of the NCP may not adequately address the diverse needs of RDNs. While RDNs with fewer years of practice may be willing to implement NCP/T, they may still find it difficult to stay motivated if they do not see the immediate benefits or if they believe the process to be complicated or time-consuming. Clinical nutrition managers and other leaders should continue to encourage the use of NCP/T, highlighting the benefits these processes bring to the organization and the nutrition profession.

#### 4.2.3 Barrier 3: lack of financial support

The association between years of practice and lack of financial support shows that depending on the career stage, RDNs may be affected differently by financial restrictions. The post-hoc analysis showed that lack of financial resources was reported significantly more often by RDNs with 6–15 years and ≥16 years of practice compared with those in the 0–5-year group. This pattern suggests that financial constraints become more salient as dietitians advance in their careers, potentially reflecting greater responsibility for securing continuing education, professional memberships, specialty training, or practice resources without institutional support. Early-career RDNs may benefit from employer-subsidized training, student-linked resources, or structured onboarding programs, whereas mid and late-career RDNs may face increasing out-of-pocket costs to maintain competency and adapt to evolving practice standards. These findings indicate that financial support mechanisms should not be limited to early career stages but should be sustained across the professional lifespan to promote continued engagement with NCP/T. Maillet et al. (16) emphasized the importance of having financial resources to ensure that RDNs can fully engage with the NCP framework. According to them, financial constraints can impede professional growth and the consistent implementation of best practices.

#### 4.2.4 Barrier 4: lack of training, education, and knowledge

The strong relationship shown between years of experience and lack of knowledge, training, and education (*p* < 0.001) implies that continuing professional development is essential to retaining skills in using NCP/T. Because NCP/T was developed more recently, RDNs who completed their education several years ago may not have received adequate training on its usage. These RDNs might not have access to further education and training, which could result in a decreased application of NCP/T. This barrier, which was reported by 29.8% of RDNs practicing for over 16 years, emphasized the value of ongoing professional development and the requirement for training courses that RDNs can enroll in at any point in their careers. According to Partlow et al. (22), continuing education provides both economic and noneconomic benefits to RDNs and the institutions they work for. Therefore, managers setting aside time and funding to improve the professional development of their staff is critical.

The finding that RDNs with 6–15 years and ≥16 years of experience reported significantly higher barriers than early-career RDNs suggests that resistance to NCP/T use may be driven less by lack of exposure and more by entrenched practice patterns and structural constraints. RDNs entering the workforce in the past 5 years are more likely to have been formally trained in NCP/T and electronic documentation systems, which may explain their lower reports of lack of knowledge, training, and motivation. In contrast, more experienced practitioners may face greater cognitive and workflow burden associated with changing long-standing documentation practices, particularly in settings with high workload demands and limited institutional support.

### 4.3 Enablers based on area of practice

#### 4.3.1 Enabler 1: requirement by the academy

The recommended use of NCP/T by a professional dietetic association was the most common enabler across all RDNs in the INIS study, clinical and community. This recommendation signifies the influential role of professional organizations in promoting standardized practices within the field. In the Canadian study, the researchers noted that 55.9% of RDNs utilized NCP/T because it was a requirement by their professional dietetic association (4). In this current study, this enabler was commonly reported by clinical (67.2%) and community (49.3%) RDNs. Notably, however, our results were stratified based on area and years of practice.

#### 4.3.2 Enabler 2: peer support

Peer support was identified as a key enabler for the use of NCP/T across practice areas among RDNs in the INIS study. Consistent with these findings, Middeke et al. (23) demonstrated that peer support is an independent predictor of both higher NCP/T knowledge and more frequent use. Together, these findings suggest that collaboration, guidance, and shared experiences with colleagues reinforce consistent application of the NCP/T and provide practical strategies for overcoming implementation barriers. This underscores the importance of social support within professional networks and suggests that fostering peer connections may strengthen confidence and facilitate routine use of the NCP/T across diverse practice settings.

#### 4.3.3 Enabler 3: electronic health records

Integration of NCP/T into EHRs was another frequently reported enabler by RDNs in the INIS study and those in the focus groups. As noted in earlier research (6), embedding NCP/T into EHR systems streamline documentation, reduces cognitive burden, and encourages adherence to standardized care processes. This system-level support can simplify workflow and promote consistent implementation, indicating that efforts to enhance EHR functionality and integration may be an effective strategy to increase the use of NCP/T across both clinical and community practice environments.

#### 4.3.4 Enabler 4: teach NCP to students

For RDNs who are dietetic educators in academia, the key enabler was the necessity of understanding NCP/T to teach it to students effectively. The highest report of this enabler was made by clinical RDNs in the INIS data (47.8%). These RDNs are often required to teach and serve as preceptors for interns. This finding emphasizes how important educators are to the continued usage of NCP/T among RDNs in future generations. Academic instructors bear the dual responsibility of ensuring that newly graduated students are adequately equipped to apply NCP/T in their future practice, in addition to improving their proficiency with these frameworks.

### 4.4 Enablers based on years of practice

#### 4.4.1 Enabler 1: requirement by the job

Among RDNs in the focus groups, this enabler was mainly reported by those with over 10 years of experience. However, this was more common among entry-level RDNs (55.2%) in the INIS data. This suggests that the institutionalization of NCP/T within professional roles is a key driver for its continued use among seasoned RDNs. This result also highlights the significance of organizational support in advancing standardized treatment procedures. Some RDNs in the focus group discussion adopted NCP/T because it is needed for CMS coding and was also incorporated into their annual performance evaluation. Usage based on employer’s mandate was consistent with findings from the largest Canadian study that examined barriers and enablers to using NCP/T (4). Managers can hone into this enabler by incorporating the use of NCP/T into performance appraisals.

#### 4.4.2 Enabler 2: regular education

In the focus groups, RDNs with over 16 years of experience in the education category identified regular education and training as key enablers. However, this was reported most frequently by entry-level RDNs (45.8%) in the INIS data. This finding reflects a strong association (*p* < 0.001) between years of practice and regular education as an enabler. This finding aligns with the broader literature on the necessity of lifelong learning to preserve the knowledge and skills in using NCP/T (24, 25). Among RDNs in the focus group discussion regular education was key. However, many requested the education to be free without any subscriptions, referring to the Academy’s webinars and subscription to eNCPT. Additionally, education must be targeted to their needs to yield maximum benefits (25).

#### 4.4.3 Enabler 3: having dedicated time to practice

Having dedicated time to practice was a significant enabler for RDNs with less than 5 years of experience (49.7%). Lövestam et al. (20) recorded this enabler among the general populations of RDNs from 10 different countries. The correlation found in this study suggests that newer RDNs may need more time to properly incorporate NCP/T into their workflows. This enabler highlights the value of encouraging work environments that can help employees adjust to the learning curve that comes with using NCP/T.

#### 4.4.4 Enabler 4: helps with communication

For clinical and community RDNs with less than 10 years of experience in the focus groups, the primary enabler was the improvement in communication facilitated by NCP/T use. This finding highlights the critical role of NCP/T in enhancing interdisciplinary communication, which is particularly valuable for new RDNs still developing their collaborative and professional routines.

#### 4.4.5 Enabler 5: peer support

Peer support was shown to be an equally significant enabler for entry-level RDNs (60.3%) from the INIS study. This support system fosters a collaborative environment where entry-level RDNs can share experiences, seek advice, and gain confidence in their skills. By interacting with their peers, they can share best practices and overcome obstacles together, enabling them to fulfill the expectations of their roles. Furthermore, the support and direction provided by seasoned RDNs can alleviate feelings of isolation and uncertainty, ultimately contributing to enhanced job satisfaction and performance among entry-level RDNs (25).

The post-hoc analyses revealed a consistent career-stage gradient across nearly all enablers, with early-career RDNs reporting the highest levels of organizational, technological, and professional supports, and late-career RDNs reporting the lowest. This suggests that system-level drivers of NCP/T use, such as EHR integration, managerial expectations, formal training, and professional guidance, are most strongly embedded during the early years of professional practice and weaken over time. These findings align with theories of professional socialization, whereby early-career clinicians are more closely aligned with institutional norms and accountability structures, while more experienced practitioners may rely more heavily on established personal workflows.

### 4.5 Strengths and limitations

A key strength of this study was the large sample size of the INIS study, which enhances the generalizability of findings to RDNs across the U.S. The focus group discussions added valuable contextual insights, such as personal experiences and attitudes, not captured in quantitative data. While not designed for statistical power, the focus groups helped identify areas for future research. Additionally, the geographical diversity of participants, representing 8 of the 9 major U.S. Regions, broadens the findings’ applicability by reflecting varied regional perspectives and practices.

Although this study aimed to capture broad, diverse, subjective data from RDNs, the data from the focus group discussions were limited to those motivated to participate which could have resulted in participation bias as RDNs who chose to participate may have stronger opinions or more familiarity with the topic compared to those who opted not to engage, leading to less representative results. Also, the participants may have been subjected to “group think” if there was a dominant RDN in their group thereby resulting in generalizability limitation.

## 5 Conclusion

This study aims to identify barriers and enablers to NCP/T use to better understand how adoption can be improved, and to assess whether qualitative feedback from practicing RDNs aligns with quantitative findings from the 2017 INIS survey (5, 20). Although the INIS study and the focus group discussions were conducted 6 years apart, both revealed consistent barriers and enablers that impact the use of NCP/T among RDNs. This consistency over time highlights the persistence of these challenges and the critical importance of addressing them to improve the adoption of NCP/T. The findings highlight the importance of designing the Academy’s training programs to address recurring barriers that vary based on RDNs’ areas of practice and years of experience. Additionally, these programs should leverage the identified enablers to facilitate implementation.

By tailoring interventions to these specific factors, the Academy can better support RDNs in adopting NCP/T in their practice, ultimately improving the quality of care and the generation of outcomes data. For example, the Academy can help address time constraints, a major barrier for RDNs in clinical settings, by advocating for EHRs that are pre-loaded with NCP/T templates, prompts, and decision-support tools. Integrating these resources directly into existing workflows reduces the time required for documentation while making it easier for RDNs to apply NCP consistently. In addition, the Academy could provide NCP/T-related resources at no additional cost to members, ensuring that every RDN has immediate access to practical guides, examples, and quick-reference tools. Together, these efforts would reduce documentation burden, support high-quality standardized practice, and promote wider adoption of NCP/T across care settings.

Also, developing concise, on-demand, evidence-based training modules that fit into busy schedules could help reduce barriers related to limited knowledge and training. Likewise, offering mobile-friendly resources and offline training options could enhance accessibility and address the barrier of limited access to online tools. Building on the findings of this study, future research should focus on developing and evaluating targeted interventions that address the specific barriers and enablers to use NCP/T across different practice areas and career stages. Given the differences in the needs of seasoned and entry-level RDNs, studies could examine the effects of tailored digital tools, time management training, and workload changes on NCP/T adoption. Longitudinal research should investigate the direct effects of NCP/T training on patient outcomes and the sustainability of changes in practice behaviors.

## Data Availability

The raw data supporting the conclusions of this article will be made available by the authors, without undue reservation.
