# Supplementary material for: Exploration of barriers and enablers for the use of the nutrition care process among a diverse sample of registered dietitian nutritionists: a mixed methods analysis
Source: Front Nutr. 2026 Jan 29;13:1727518. doi: 10.3389/fnut.2026.1727518 (PMC12894035; doi:10.3389/fnut.2026.1727518)
Supplement: Supplementary file 1 [file Data_Sheet_1.docx]

Supplementary Material

# Focus Group Interview Guide – Clinical

Introduction:

Hello, I am Casey Colin and I am interviewing you as part of a study to gather insights regarding the Nutrition Care Process and use of Nutrition Care Process Terminology. No information identifying you will be asked or collected during this interview. I will ask you twenty-three questions; please feel free to speak as candidly and openly as possible when answering each question. The interview will be recorded only for transcription purposes. Your responses will be transcribed for use in my research, but otherwise this recording will not be shared with anyone. Are you ready for me to ask the first question?

Opening Question:

Let’s start with a fun question:

- What is your favorite way to unwind after a day of work?

Introductory Questions:

- How many years have you been an RDN, and how many years have you been in your current role?
- What is your current role, and what is your favorite part of that role?

Transition Question:

- How were you first introduced to the NCP?

Key Questions:

Attitudes

- What are your opinions on the use of the NCP in dietetics practice?
- What are your opinions on the use of the NCPT in dietetics documentation?

Behavioral intention

- What has influenced you to use or not use the NCP in dietetics practice?
- What has influenced you to use or not use the NCPT in dietetics documentation?

Subjective norms

- What do you think other RDNs feel about the NCP?
- In what settings do you think the NCP is more likely to be used?
- What do you think other RDNs feel about the NCPT?
- In what situations do you think NCPT is more likely to be used?

Social norms

- What aspects of the NCP do you think are followed most often in dietetics practice?
- How would you describe “usual” MNT documentation?
- How often do you think RDNs are using the NCPT in MNT documentation?
- What aspects of the NCPT do you think are most often used in MNT documentation?

Perceived power

- How would you say your knowledge of the NCP has influenced you to use or not use it in practice?
- How would you say your knowledge of the NCPT has influenced you to use or not use it in practice?
- How have you felt forced to use NCP/T?

Perceived behavioral control

- What would make the NCP more widely utilized by RDNs?
- What would make the NCPT more widely utilized by RDNs?
- How could the Academy influence use of NCP and NCPT among RDNs?
- What are other factors which could influence use of NCPT and NCPT among RDNs?

Closing Remarks

This concludes the focus group. The Zoom call will end in a moment. Thank you so much for taking the time to participate, and have a great rest of your day!

# Focus Group Interview Guide – Community

Introduction:

Hello, I am Casey Colin and I am interviewing you as part of a study to gather insights regarding the Nutrition Care Process and use of Nutrition Care Process Terminology. No information identifying you will be asked nor collected during this interview. I will ask you twenty-three questions; please feel free to speak as candidly and openly as possible when answering each question. The interview will be recorded only for transcription purposes. Your responses will be transcribed for use in my research, but otherwise this recording will not be shared with anyone. Are you ready for me to ask the first question?

Opening Question:

Let’s start with a fun question:

- What is your favorite way to unwind after a day of work?

Introductory Questions:

- How many years have you been an RDN, and how many years have you been in your current role?
- What is your current role, and what is your favorite part of that role?

Transition Question:

- How were you first introduced to the NCP?

Key Questions:

Attitudes

- What are your opinions on the use of the NCP in dietetics practice?
- What are your opinions on the use of the NCPT in dietetics documentation?

Behavioral intention

- What has influenced you to use or not use the NCP in dietetics practice?
- What has influenced you to use or not use the NCPT in dietetics documentation?

Subjective norms

- What do you think other RDNs feel about the NCP?
- In what settings do you think the NCP is more likely to be used?
- What do you think other RDNs feel about the NCPT?
- In what situations do you think NCPT is more likely to be used?

Social norms

- What aspects of the NCP do you think are followed most often in dietetics practice?
- How would you describe “usual” nutrition care documentation?
- How often do you think RDNs are using the NCPT in nutrition care documentation?
- What aspects of the NCPT do you think are most often used in nutrition care documentation?

Perceived power

- How would you say your knowledge of the NCP has influenced you to use or not use it in practice?
- How would you say your knowledge of the NCPT has influenced you to use or not use it in practice?
- How have you felt forced to use NCP/T?

Perceived behavioral control

- What would make the NCP more widely utilized by RDNs?
- What would make the NCPT more widely utilized by RDNs?
- How could the Academy influence use of NCP and NCPT among RDNs?
- What are other factors which could influence use of NCPT and NCPT among RDNs?

Closing Remarks

This concludes the focus group. The Zoom call will end in a moment. Thank you so much for taking the time to participate, and have a great rest of your day!

## 3. Focus Group Interview Guide – Education

Introduction:

Hello, I am Casey Colin and I am interviewing you as part of a study to gather insights regarding the Nutrition Care Process and use of Nutrition Care Process Terminology. No information identifying you will be asked nor collected during this interview. I will ask you twenty-three questions; please feel free to speak as candidly and openly as possible when answering each question. The interview will be recorded only for transcription purposes. Your responses will be transcribed for use in my research, but otherwise this recording will not be shared with anyone. Are you ready for me to ask the first question?

Opening Question:

Let’s start with a fun question:

- What is your favorite way to unwind after a day of work?

Introductory Questions:

- How many years have you been an RDN, and how many years have you been in your current role?
- What courses do you teach, and which is your favorite?

Transition Question:

- How were you first introduced to the NCP?

Key Questions:

Attitudes

- What are your opinions on the use of the NCP in dietetics practice?
- What are your opinions on the use of the NCPT in dietetics documentation?

Behavioral intention

- What has influenced how you teach the NCP in dietetics education?
- What has influenced how you teach the NCPT in dietetics education?

Subjective norms

- What do you think other RDNs feel about the NCP?
- In what settings do you think the NCP is more likely to be used?
- What do you think other RDNs feel about the NCPT?
- In what situations do you think NCPT is more likely to be used?

Social norms

- What aspects of the NCP do you think are most often taught in dietetics education?
- How would you describe “usual” nutrition care documentation?
- How often do you think RDNs are teaching the NCPT in dietetics education?
- What aspects of the NCPT do you think are most often taught in dietetics education?

Perceived power

- How would you say your knowledge of the NCP has influenced how you teach it?
- How would you say your knowledge of the NCPT has influenced how you teach it?
- How have you felt forced to teach NCP/T?

Perceived behavioral control

- What would make the NCP more thoroughly taught in dietetics education?
- What would make the NCPT more thoroughly taught in dietetics education?
- How could the Academy influence how NCP and NCPT are taught in dietetics education?
- What are other factors which could influence how NCP and NCPT are taught in dietetics education?

Closing Remarks

This concludes the focus group. The Zoom call will end in a moment. Thank you so much for taking the time to participate, and have a great rest of your day!

**
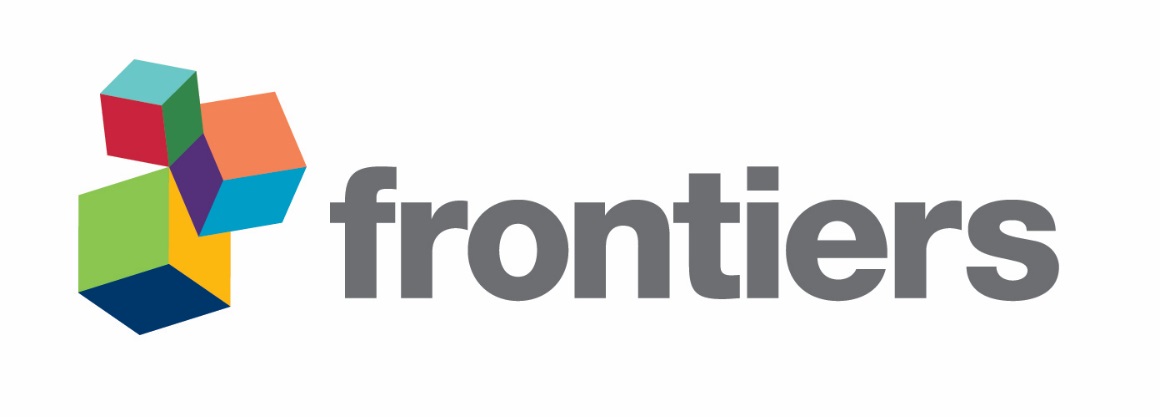
**
